# Editorial: Oscillatory brain activity as a marker of brain function and dysfunction in aging and in neurodegenerative disorders

**DOI:** 10.3389/fnagi.2023.1153150

**Published:** 2023-02-15

**Authors:** Aneta Kielar, Priyanka Shah-Basak, Lars Meyer, Takako Fujioka

**Affiliations:** ^1^Speech Language and Hearing Sciences, University of Arizona, Tucson, AZ, United States; ^2^Department of Neurology, Medical College of Wisconsin, Milwaukee, WI, United States; ^3^Max Planck Institute for Human Cognitive and Brain Sciences, Leipzig, Germany; ^4^Clinic for Phoniatrics and Pedaudiology, University Hospital Münster, Münster, Germany; ^5^Department of Music, Stanford University, Stanford, CA, United States

**Keywords:** neural oscillations, healthy aging, neurodegenerative disorders, biomarkers of neurodegeneration, oscillatory slowing

*Neural oscillations* support fundamental mechanisms of information processing in neural networks (Buzsáki, 2006; Singer, 2013, 2018). Properties such as phase and amplitude during task performance and at rest map onto cognitive processes and abilities. The articles in this issue discuss the scientific and clinical use of oscillations in neurological populations that exhibit altered cognition. The articles focus on understanding the oscillatory changes associated with healthy aging and progression of neurodegenerative disorders such as Alzheimer's Disease (AD), Mild Cognitive Impairment (MCI) and Parkinson's Disease (PD), and examine how oscillatory activity can inform development of interventions to slow down aging-related cognitive decline. Nine exciting contributions provide novel methods involving oscillatory signals as early indicators of healthy aging, biomarkers of neurodegeneration, and predictors of successful interventions.

## Oscillatory changes associated with healthy aging

Griffa et al. investigated oscillatory connectivity, derived from resting-state MEG (rsMEG) signals in cognitively impaired and normal oldest-old adults (90+ years old) in terms of its relationships to their cognitive status. In the impaired oldest-old participants, increased theta but reduced beta power was found in frontoparietal regions and the default mode network, indicative of cortical slowing. Engagement during demanding cognitive tasks (indicator of cognitive reserve) was associated with stronger connectivity in the alpha and beta bands. Overall, the oscillatory changes in the oldest-old could not be readily distinguished from individuals younger than 85 years.

Fröhlich et al. examined differences in spectral power and oscillatory reactivity in 80+-year old adults across different cognitive status (cognitively unimpaired, possible MCI, non-amnesic MCI, amnesic MCI). No differences in power during eyes-closed condition were found between healthy individuals and those with cognitive impairments. The authors noted that these findings might be related to anatomical changes associated with advanced aging such as cortical thinning which could lower baseline EEG amplitudes. Although this was not directly addressed in the study, the report highlights the importance of additional anatomical information in this population to reliably interpret scalp-level oscillations.

## Changes in the EEG signal associated with neurodegeneration

Doan et al. examined the utility of resting-state and sensory and task-related EEG measures to predict dementia severity based on MMSE scores. After adjusting for demographic confounds, prefrontal EEG measures were found to be highly correlated with MMSE. Furthermore, relationships within EEG measures, including peak frequency, median frequency, alpha-to-theta ratio, alpha asymmetry, and theta-band power indicated increased risk of dementia. This preliminary evidence suggests a potential role of rsEEG as a screening tool. But larger-scale studies will need to substantiate these findings with cautious study designs, matching dementia and non-dementia groups on important demographic variables.

Smailovic et al. investigated the relative potential of rsEEG power, rsEEG connectivity and novel (neurogranin) and conventional CSF markers (amyloid and tau pathology) to differentiate subtypes of amnestic MCI. The strongest discriminator between single-domain and multi-domain MCI was a connectivity measure—global synchrony—in theta and delta bands. Connectivity in slow frequencies was related to early effects of AD-specific molecular pathology, further promising the utility of rsEEG measures as potential biomarkers of dementia.

Lopez et al. modeled connectivity hubs from resting-state alpha measurements in AD. Although both controls and individuals diagnosed with AD had significant parietal “degree” and “connector” hubs derived from alpha rhythms, outward directionality of parietal hubs was lower in AD.

## Oscillatory power as a predictor of successful non-pharmacological and pharmacological interventions

Spironelli and Borella examined short- and long-term effects of working memory (WM) training among healthy older adults on behavioral and rsEEG-based oscillatory indices. Specific training, maintenance and transfer effects were reported in the WM treatment group compared to the active control group. For oscillatory responses, the treatment group showed increased oscillatory responses in bilateral anterior sites, which were correlated with better post-training performance.

Complementary to the assessment of resting-state power, Rodríguez-González et al. implemented a network-based approach that considered complex interactions among neurophysiological, cognitive (MMSE), and behavioral (Dementia Behavior Disturbance Scale) variables to assess treatment outcomes. Together the results indicate that changes in EEG parameters can serve as indicators of treatment-related changes in cognition and behavior.

Eyjolfsdottir et al. examined the effects of targeted encapsulated cell biodelivery of nerve growth factor (NGF-ECB) in the basal forebrain (a treatment approach for AD) on rsEEG parameters and cognition over a 12-month period. Increased theta power was associated with a decrease in CSF cholinergic marker (ChAT), whereas increased alpha power was related to increased ChAT and stabilization of MMSE scores.

Zhang et al. examined rsEEG oscillatory and non-oscillatory changes induced by dopaminergic medication in patients with PD. Beta-band phase synchronization was up-regulated by medication. Medication also increased the spectral slope of broadband non-oscillatory component, suggesting that spectral slope could serve as a marker of global efficiency.

In summary, cognitive impairment is found to be associated with increases in slow frequency and reduction in higher frequency cortical rhythms. Oscillatory reactivity is also modulated by behavioral and pharmacological interventions, and can serve as an indicator of treatment success alongside with other biomarkers. All studies here examined rsMEEG measures mainly including spectral power, ratio between bands, shifts in bands, and connectivity using phase- or amplitude-based metrics, suggesting possible mechanistic roles of oscillations.

## Future directions

This Research Topic provides new methodologies and results with a potential to advance research and clinical practice for aging populations. Most reported results are preliminary, and signal a need for robust, well-designed, large-scale clinical trials with a focus on spectral oscillatory measures to replicate the said findings. Longitudinal studies are needed to validate reliability in predicting cognitive status. Specific to connectivity, approaches that overcome known challenges regarding volume conduction, linear mixing, and signal-to-noise ratio should be used (Palva and Palva, 2012). Consistent and reproducible findings related to connectivity are needed to better interpret normal and pathological oscillatory dynamics (Colclough et al., 2015; van Diessen et al., 2015).

## Author contributions

AK: conceptualization, writing, review, and editing. PS-B and LM: writing, review, and editing. TF: review and editing. All authors contributed to the article and approved the submitted version.
